# Indirect estimation of serum enzymes reference intervals in adults using the reflimR and refineR algorithms

**DOI:** 10.11613/BM.2026.010706

**Published:** 2026-02-15

**Authors:** Raziye Yıldız, Fatma Demet Arslan, Mehmet Köseoğlu

**Affiliations:** Department of Medical Biochemistry, Faculty of Medicine, Bakırcay University, Izmir, Turkey

**Keywords:** reference ranges, big data, clinical enzyme tests, laboratory information systems

## Abstract

**Introduction:**

Many clinical laboratories rely on manufacturer-provided reference intervals (RIs) because of logistical and financial constraints of direct RI estimation. Indirect estimation methods offer a practical alternative for deriving RIs from laboratory data. This study aimed to estimate RIs for eight serum enzymes using the R-based algorithm reflimR, and to compare them with refineR, manufacturer’s instructions for use (IFU), and direct methods.

**Materials and methods:**

Data from adult outpatients tested between January 2021 and May 2022 were retrospectively analyzed for alkaline phosphatase (ALP), alanine aminotransferase (ALT), amylase, aspartate aminotransferase (AST), creatine kinase (CK), gamma-glutamyl transferase (GGT), lactate dehydrogenase and lipase. Reference intervals were estimated using reflimR and refineR, and compared with IFU and direct RIs. Overlap between lower and upper limits was evaluated using a color-coded scheme. Data distribution was tested with Shapiro–Wilk; and Mann–Whitney U and Spearman’s correlation tests were used for group comparisons and correlations.

**Results:**

Sex-specific RIs were required for ALP, ALT, AST, CK and GGT. ReflimR generally produced wider intervals than refineR. Agreement of reflimR with refineR, parametric, and IFU-based RIs was 88.5%, 72.7%, and 62.5%, respectively. The lowest agreement was observed with the non-parametric method (55.0%).

**Conclusions:**

ReflimR provides a practical approach for indirect RIs estimation from routine data. Its performance was comparable to refineR and parametric methods, supporting its use for verifying or updating local RIs, especially where population-specific RIs are unavailable. To our knowledge, this is the first study to apply reflimR to the Turkish population and directly compare its performance with refineR and IFUs.

## Graphical abstract

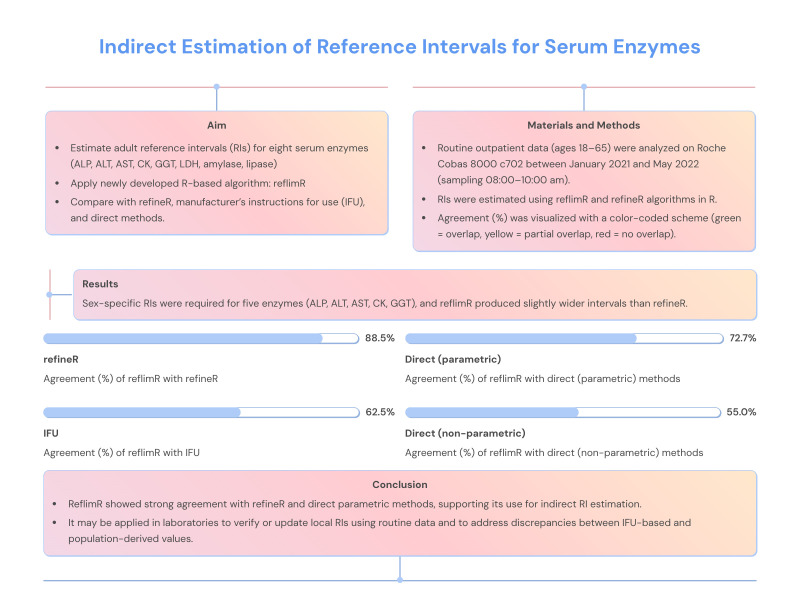

## Highlights

• Reference intervals were estimated using an indirect method

• Eight serum enzymes were analyzed: ALP, ALT, AST, CK, GGT, LDH, amylase, lipase

• ReflimR and refineR showed high agreement (88.5%)

• Sex-specific reference intervals were needed for ALP, ALT, AST, CK, GGT

• ReflimR provides a practical tool for reference interval verification

## Introduction

Reference intervals (RIs), typically defined as the central 95% of laboratory test results obtained from a healthy population, are essential tools for the clinical interpretation of laboratory data (*1*). However, RIs can vary considerably depending on factors such as sex, age, ethnicity, geographic region, and lifestyle. Therefore, international guidelines recommend the use of population-specific RIs to ensure accurate clinical decision-making (*1*, *2*).

Traditionally, RIs have been established using the direct method, which involves recruiting healthy volunteers and performing controlled measurements to define the reference distribution. Although this method is considered the gold standard, it is often limited by substantial logistical, financial, and time-related constraints, making it impractical for many laboratories (*3*, *4*).

To address these limitations, indirect statistical methods have been developed to estimate RIs from existing patient data within laboratory information systems. These methods aim to isolate results that reflect non-pathological distributions, thereby enabling RI estimation without the need for dedicated sampling (*3*, *5*). Notably, such methods benefit from large sample sizes and reflect the diversity observed in routine clinical data, which may better capture physiological variability (*6*, *7*). International recommendations, including the Clinical and Laboratory Standards Institute guideline (EP28-A3c) and the International Federation of Clinical Chemistry and Laboratory Medicine Committee on Reference Intervals and Decision Limits (C-RIDL), also endorse indirect approaches as a practical alternative for establishing RIs (*1*, *3*).

Among the available indirect estimation methods, refineR and reflimR are freely accessible R-based algorithms specifically designed for RI estimation. The refineR algorithm applies an inverse modelling approach, scanning data segments, identifying potential cut-off points, and applying Box-Cox transformation with parameter fitting to estimate the non-pathological distribution (*8*). In contrast, reflimR, assumes either a normal or log-normal distribution and employs a trimming strategy based on boxplot thresholds, from which reference limits are derived using a truncated quantile-quantile plot (*6*, *9*).

Most laboratory test results, including enzyme activity assays, do not follow a normal distribution. Enzymes frequently exhibit right-skewed distributions due to their asymmetric physiological behavior, making them particularly suitable for modeling with indirect methods that accommodate non-Gaussian data structures (*1*, *7*).

This study aimed to estimate adult RIs for eight commonly measured serum enzymes using the newly developed reflimR algorithm, and to compare its performance with refineR, manufacturer’s instructions for use (IFU), and previously published direct RI studies in the Turkish population. We hypothesized that reflimR would yield RIs comparable to those obtained by refineR and direct methods, while providing more population-representative values than IFUs.

## Materials and methods

This retrospective study included routine laboratory test results analyzed from blood samples collected between 8 am and 10 am from adult outpatients (aged 18-65 years) at a University Hospital in Western Turkey between January 2021 and May 2022. The study protocol was approved by the Ethics Committee of Bakırçay University Faculty of Medicine (Approval No: 2151, dated March 19, 2025).

Serum enzyme measurements included alkaline phosphatase (ALP, U/L), alanine aminotransferase (ALT, U/L), amylase (U/L), aspartate aminotransferase (AST, U/L), creatine kinase (CK, U/L), gamma-glutamyl transferase (GGT, U/L), lactate dehydrogenase (LD, U/L), and lipase (U/L). All tests were performed on the Cobas 8000 c702 analyzer (Roche Diagnostics, Mannheim, Germany) using enzymatic colorimetric methods according to manufacturer protocols. The analytical limit of detection values provided by the manufacturer were as follows: ALT, AST, and ALP were 5 U/L; GGT, amylase, and lipase were 3 U/L; CK was 7 U/L; and LD was 10 U/L. Internal and external quality control (QC) procedures were implemented throughout the study period. Internal QC was performed daily using two levels of commercial control materials (PreciControl ClinChem Multi 1 and 2, Roche Diagnostics, Mannheim, Germany), and results were monitored according to Westgard rules. During the study period, the coefficients of variation were < 5% for all enzymes. External QC was ensured through participation in the Randox International Quality Assessment Scheme (RIQAS) program, with monthly proficiency testing, all of which were within acceptable limits.

Only the first test result *per* patient was included. Records with missing data or values outside the analytical limit of detection were excluded. Outliers were removed based on Tukey’s rule. Data distribution was assessed using the Shapiro–Wilk test and visual inspection of histograms. Because all enzyme activity values were not normally distributed, sex-based differences were assessed using the Mann–Whitney U test, and correlations between age and enzyme values were evaluated using Spearman’s rank correlation.

Reference intervals were estimated using two indirect methods: refineR (v1.6.2) and reflimR (v1.0.6), both implemented in R software version 4.3.3 (R Foundation for Statistical Computing, Vienna, Austria; https://www.r-project.org/). Following previous evaluations of indirect methods, a minimum sample size of > 200 was considered adequate for reflimR and > 1000 for refineR to ensure robust estimation (*6*). Estimates were compared with RIs provided in IFUs, based on Roche Diagnostics reagents. These values were also compared against parametric and non-parametric RIs reported in direct studies of the Turkish population (*10*).

The agreement between RI estimation methods for the lower limit (LL) and upper limit (UL) of each enzyme was assessed with the tolerance range approach using permissible uncertainty function from the reflimR package. The 95% confidence intervals (CIs) for LL and UL were estimated with precomputed Monte Carlo–based closed formulas the using conf_int95 function from the reflimR package (*9*). A traffic light visualization was used to illustrate agreement: green means the target value is within the reflimR tolerance range, yellow means the target value is outside but the tolerance ranges overlap, and red means the tolerance ranges are completely separate (*6*).

## Results

Significant sex-related differences were observed for ALP, ALT, AST, CK, and GGT, whereas LD, amylase, and lipase showed no relevant differences; therefore, sex-specific RIs were estimated only for these analytes. Spearman’s correlation analyses revealed no relevant correlation between age and enzyme values. Although statistically significant due to the large sample size, all observed coefficients were negligible (r < 0.25, P < 0.001 for all).

**Figure 1:**
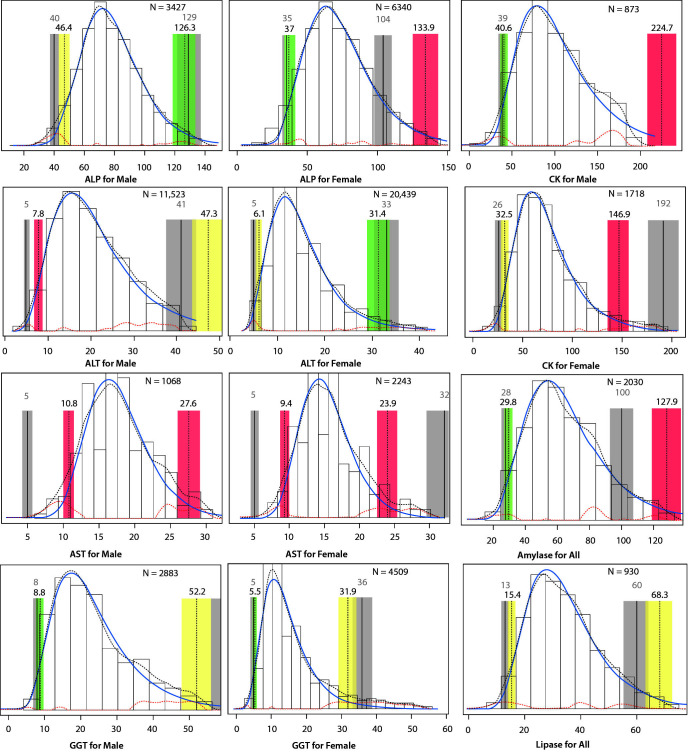
Comparison of reference intervals (Ris, IU/L) estimated by reflimR with manufacturer's instructions for use (IFU). The black and red dashed density curves represent the distributions of the assumed reference population and potential pathological outliers, respectively. The solid blue line indicates the fitted curve to the assumed distribution, and the background histogram represents the frequency distribution of routine data. Vertical dashed lines show the lower limit (LL) and upper limit (UL) estimated by reflimR, while vertical solid lines show IFU values. The shaded areas around the vertical lines represent tolerance ranges based on permissible uncertainty. Agreement is illustrated using a traffic-light color code: green indicates that the target limit is within the tolerance range of the limit estimated by reflimR; yellow indicates that the target limit is outside the tolerance range of the estimated limit, but the tolerance ranges of the target and estimated limits overlap; and red indicates that the tolerance ranges of the target and estimated limits do not overlap. ALP - alkaline phosphatase. ALT - alanine aminotransferase. AST - aspartate aminotransferase. CK - creatine kinase. GGT - gamma-glutamyl transferase.

Tables 1 and 2 summarize the RIs obtained from IFUs, direct methods (parametric and non-parametric), and indirect methods (reflimR and refineR). It also includes sample sizes and information regarding sex-specific partitioning. Figure 1 shows a graphical comparison between the RIs estimated by reflimR and those provided in the IFUs.

**Table 1:** Reference intervals for enzymes with sex-specific partitioning

			**Reference range derived by reflimR**		**Reference range derived by refineR**		**Reference range given in IFU**		**Parametric**		**Non-parametric**	
**Para-** **meters** **(U/L)**	**Sex**	**N**	**LL** **(95% CI)**	**UL** **(95% CI)**	**LL** **(95% CI)**	**UL** **(95% CI)**	**LL**	**UL**	**LL** **(90% CI)**	**UL** **(90% CI)**	**LL** **(90% CI)**	**UL** **(90% CI)**
ALP	M	3685	46.4 (45.3-48.0)	126.3 (122.1-129.4)	44.4^†^ (39.1-47.8)	115^†^ (90.7-131)	40^†^	129*	43^†^ (40-46)	116^†^ (113-120)	42^†^ (41-44)	120* (116-123)
	F	6615	37.0 (36.1-38.2)	133.9 (129.8-137.0)	37.2* (36.3-40.1)	132* (118-135)	35*	104^‡^	35^†^ (34-38)	105^‡^ (102-111)	36* (34-37)	110^‡^ (106-115)
ALT	M	11799	7.8 (7.61-8.06)	47.3 (45.79-48.47)	7.85* (7.17-7.98)	46.3* (32.3-47.4)	5^‡^	41^†^	9^†^ (8.2-9.2)	57^‡^ (53.3-61.9)	8* (8.0-9.0)	58^‡^ (55.0-61.0)
	F	22293	6.1 (6.00-6.23)	31.4 (30.7-31.91)	6.09* (4.81-6.18)	28.2^†^ (20.6-30.7)	5^†^	33*	7^†^ (6.8-7.2)	28^†^ (26.0-29.2)	7^†^ (6.0-7.0)	33* (29.0-35.1)
AST	M	1247	10.8 (10.4-11.5)	27.6 (26.0-28.7)	9.25^†^ (8.66-10.7)	25.1^†^ (23.9-28.5)	5^‡^	40^‡^	13^‡^ (12.6-13.3)	30^†^ (29.7-31.7)	13^‡^ (12.0-13.0)	36^‡^ (34.0-38.0)
	F	2591	9.4 (9.15-9.77)	23.9 (23.01- 24.55)	8.66^†^ (8.34-9.45)	22.8^†^ (20.9-25.8)	5^‡^	32^‡^	11^‡^ (10.5-11.1)	25* (24.3-26.5)	11^‡^ (10.0-11.0)	28^‡^ (26.1-29.2)
CK	M	902	40.6 (37.4-46.2)	224.7 (197.4-244.1)	39.3* (23.2-58.1)	209* (132-232)	39*	308^‡^	48^†^ (45-54)	227* (221-248)	47^†^ (43-49)	252^†^ (239-266)
	F	1849	32.5 (30.9-35.0)	146.9 (136.3-154.6)	31.5* (28-34)	121^†^ (106-143)	26^†^	192^‡^	34* (30-36)	131^†^ (116-139)	32* (27-34)	135^†^ (126-151)
GGT	M	3110	8.8 (8.40-9.40)	52.2 (48.88-54.70)	9.14* (7.87-9.33)	43.7^‡^ (37.3-47.4)	8*	61^†^	11^†^ (10.1-11.9)	70^‡^ (68-77)	11^†^ (10.0-11.0)	78^‡^ (69-82)
	F	5062	5.5 (5.30-5.78)	31.9 (30.4-33.08)	5.96* (5.4-6.1)	24.3^‡^ (19.5-30.0)	5*	36^†^	7^‡^ (7.0-7.7)	33* (31-37)	7^‡^ (7.0-8.0)	39^‡^ (36-44)
IFU - manufacturer’s instructions for use. Parametric and non-parametric - reference intervals from direct method studies in the literature. *The target limit is within the tolerance range of the limit estimated by reflimR. ^†^The target limit is outside the tolerance range of the estimated limit, but the tolerance ranges of the target and estimated limits overlap. ^‡^The tolerance ranges of the target and estimated limits do not overlap. LL - lower reference limit. UL - upper reference limit. M - male. F - female. ALP - alkaline phosphatase. ALT - alanine aminotransferase. AST - aspartate aminotransferase. CK - creatine kinase. GGT - gamma-glutamyl transferase.												

**Table 2:** Reference intervals for enzymes with unified partitioning

		**Reference range derived by reflimR**		**Reference range derived by refineR**		**Reference range given in IFU**		**Parametric**		**Non-parametric**	
**Parameters** **(U/L)**	**N**	**LL** **(95% CI)**	**UL** **(95% CI)**	**LL** **(95% CI)**	**UL** **(95% CI)**	**LL**	**UL**	**LL** **(90% CI)**	**UL** **(90% CI)**	**LL** **(90% CI)**	**UL** **(90% CI)**
Amylase	2121	29.8 (28.5-31.9)	127.9 (119.7-134.0)	29.2* (19.6-31.2)	114^†^ (84.8-128)	28*	100^‡^	NA	NA	NA	NA
LD	5540	118 (116-120)	233 (229-236)	118* (116-120)	222^†^ (209-232)	NA	NA	126^†^ (NA)	220^†^ (NA)	NA	NA
Lipase	973	15.4 (14.4-17.2)	68.3 (61.3-73.2)	14.9* (12.1-19.9)	55.4^‡^ (41.2-68)	13^†^	60^†^	NA	NA	NA	NA
IFU - manufacturer’s instructions for use. Parametric and non-parametric - reference intervals from direct method studies in the literature. *The target limit is within the tolerance range of the limit estimated by reflimR. ^†^The target limit is outside the tolerance range of the estimated limit, but the tolerance ranges of the target and estimated limits overlap. ^‡^The tolerance ranges of the target and estimated limits do not overlap. LL - lower reference limit. UL - upper reference limit. LD - lactate dehydrogenase. NA - not available.											

Overlap between the RIs estimated by reflimR and refineR was observed for both sexes, except for the ULs of GGT and lipase. The agreement between refineR and reflimR, defined as the proportion of comparisons classified as green or yellow in the color-coded system, was 88.5% (23 out of 26) across all analytes. As shown in Tables 1 and 2, reflimR tended to yield higher ULs than refineR, which resulted in broader RIs in most analytes. The agreement between IFU-provided RIs and those estimated by reflimR was 62.5% (15 out of 24). Agreement with direct methods was 72.7% (16 out of 22) for the parametric method and 55.0% (11 out of 20) for the non-parametric method.

For ALP, although reflimR and refineR estimates showed close agreement in both sexes, reflimR produced slightly wider intervals. While the UL derived from reflimR was comparable across other methods in males, it substantially exceeded the ULs provided by IFU and direct methods in females.

For ALT and AST, reflimR produced similar RIs to those of refineR in both sexes. While reflimR in males yielded a higher but acceptable UL of ALT than IFU, the UL estimated by reflimR was significantly lower than those reported by direct methods. Instructions for use was notable for having the widest RI and highest UL for AST. While reflimR yielded a significantly lower UL for AST than that of non-parametric methods in both sexes, it was comparable to the values obtained using parametric methods.

For CK, reflimR and refineR produced similar RIs in both sexes. The IFU provided higher ULs compared to both indirect and direct methods. Notably, all methods revealed marked sex-related differences with higher ULs in males.

For GGT, indirect methods yielded lower ULs compared to IFU and direct methods. The UL estimated by reflimR was substantially higher than that of refineR in both sexes. Direct methods, especially non-parametric ones, yielded higher ULs, up to 78 U/L in males.

For amylase and lipase, reflimR estimated wider RIs than IFU. The UL tolerance limits for amylase did not overlap with those of IFU, whereas the ULs for lipase did. Since sex-based RIs for these tests were not estimated by reflimR, no comparison with direct methods was performed.

For LD, reflimR produced RIs similar to those obtained with refineR and parametric methods. Since the IFU presents sex-based RIs, they were not included in the comparison.

## Discussion

In this study, we compared adult RIs estimated by indirect methods (reflimR and refineR), direct methods (parametric and non-parametric), and IFU values for eight commonly used serum enzymes activities. ReflimR demonstrated strong agreement with refineR (88.5%) and substantial agreement with parametric methods (72.7%). The lowest agreement was found for IFU values (62.5%) and non-parametric estimates (55.0%). These findings highlight the practical utility of indirect approaches, particularly reflimR, to verify and update local RIs using patient data.

The limited agreement with IFU values likely reflects that such intervals are often derived from restricted or non-representative populations, or even from literature values, and may therefore not capture local demographic and environmental characteristics, as also reported in C-RIDL studies (*11*, *12*). In contrast, non-parametric estimates are highly sensitive to sample size and outliers; if the dataset is not sufficiently large and well-characterized, the resulting intervals may not represent the true population distribution (*11*). These methodological limitations can reduce concordance with indirect approaches. Consistent with our findings, recent multicenter and big data studies have shown that indirect and parametric methods yield more robust and comparable reference intervals, whereas IFU and non-parametric methods are more prone to variability (*12*, *13*).

Overall, reflimR produced comparable or slightly wider RIs than refineR, depending on the analyte and sex. In our analysis, the broader intervals produced by reflimR compared to refineR may be attributed to its simpler assumption of normal or log-normal distributions, rather than the more complex statistical modeling of the pathological distribution used by refineR.

Agreement between reflimR and direct methods varied by analyte, with closer overlap for ALP (males), ALT (females), and CK (both sexes), and weaker concordance for AST and GGT. Such discrepancies may reflect methodological differences as well as population-specific factors. We did not compare the RIs of amylase and lipase with direct methods because we used sex-independent intervals for these analytes.

ReflimR estimated a higher UL for ALT in males and lower ULs for AST in both sexes compared to IFU. Similarly, Özarda *et al.* reported higher ALT values in males and lower AST ULs in both sexes compared to the Abbott Diagnostics IFU values (*10*). They also observed narrower RIs for ALT and AST in females than in males. Similar to our findings, Köseoğlu *et al.* reported markedly narrower RIs for AST compared to the Abbott Diagnostics IFU values (*14*). These findings suggest sex-specific variability in ALT and AST activities, as well as discrepancies between IFU values and population-based estimates (*10*, *14*, *15*).

For ALP, reflimR produced the widest interval across all methods, with female ULs markedly higher than both IFU and previous reports (*10*). These findings suggest that reflimR may better reflect physiological diversity, particularly in females, compared to IFU.

Creatine kinase exhibited the most pronounced sex-related differences, consistent with physiological determinants such as muscle mass, hormonal influences, and physical activity (*16*, *17*). These findings reinforce the need for sex-specific RIs, while the high UL provided by the IFU may represent an overestimation relative to population-based values.

The lack of overlap between indirect and direct estimates suggests that GGT is sensitive to population-specific factors such as alcohol use and metabolic status. These results underscore the value of indirect methods in generating more conservative and population-representative intervals.

The higher ULs of amylase and lipase estimated by reflimR may more accurately reflect physiological variation. Similar findings have been reported in studies using the OPUS::L and modified Bhattacharya methods, which are both indirect methods that estimate RIs from large routine laboratory datasets. OPUS::L applies statistical models such as truncated maximum likelihood to extract non-pathological values, while the Bhattacharya method identifies the main (presumed healthy) population by fitting frequency distributions (*4*, *17*). Both methods yielded higher ULs which raise concerns about misclassification or overdiagnosis when narrow IFUs are used.

Minimal sex-related variation across methods justifies the use of a unified RI for LD. Consistent with Ozarda *et al.* and Omuse *et al.*, sex-related differences in LD activities were minimal, supporting the use of unified RIs for this analyte (*10*, *16*).

Sex appears to be a critical determinant in enzyme distribution. Significant sex-based differences were observed for ALT, AST, ALP, GGT, and CK, reinforcing the need for sex-partitioned RIs in clinical interpretation. In contrast, LD, amylase, and lipase showed minor sex-related variability, making unified RIs feasible. These findings are consistent with previous reports (*10*, *14*, *16*).

Sun *et al.* identified a need for age partitioning only for GGT in males, while no such requirement was found for ALP or AST (*18*). Some studies reported significant age-related differences, particularly ALT and ALP in females (*11*, *15*, *16*). Taken together, these observations reinforce the notion that age-related variation in enzyme activity is generally modest and tends to manifest selectively in subgroups of certain analytes. This may justify our decision not to apply age-based partitioning in this study.

The indirect methods must be highly sensitive in distinguishing non-pathological distributions in laboratory databases with a high proportion of pathological values (*8*, *19*). It has been reported that optimal performance is achieved when the pathological fraction is below 30%, with a dataset of more than 200 for reflimR and more than 1000 for refineR (*7*, *20*, *21*). In our study, all analytes, except CK in males (N = 902) and lipase (N = 973), met the recommended sample size. The pathological fraction was below 21% for all enzymes, supporting the robustness of the derived RIs.

To the best of our knowledge, this is the first study to establish enzyme RIs in the Turkish adult population using both refineR and the newly introduced R-based algorithm, reflimR. Nonetheless, several limitations should be acknowledged. The study population was limited to adults, excluding both pediatric and geriatric groups. Age-specific RIs were not established, since the correlation between age and enzyme activity was minimal. Slight increases in enzyme activities with advancing age may be confounded by factors such as medication use or increased body mass (*22*). Consequently, distinguishing between age-related pathological elevations and normal physiological changes remains a challenge in the interpretation of laboratory results. Finally, as the data were generated on a single analytical platform, the generalizability of these RIs to other platforms may be limited.

The observed discrepancies between IFU-based values and population-derived estimates emphasize the importance of establishing RIs tailored to the target population. Leveraging large-scale laboratory data to estimate population-specific RIs improves the interpretation of test results and may help reduce unnecessary clinical interventions. ReflimR may offer a practical solution for clinical laboratories with limited resources to conduct direct RI studies. In practice, laboratory specialists can verify or update their local RIs with reflimR by using routinely laboratory data, provided adequate sample size and data quality are ensured.

## Data Availability

The data generated and analyzed in the presented study are available from the corresponding author on request.
